# Nursing in the Artificial Intelligence (AI) Era: Optimizing Staffing for Tomorrow

**DOI:** 10.7759/cureus.47275

**Published:** 2023-10-18

**Authors:** Abdulqadir J Nashwan, Ahmad A Abujaber

**Affiliations:** 1 Nursing Department, Hamad Medical Corporation, Doha, QAT

**Keywords:** staff assignment, staffing, nursing shortage, nursing, artificial intelligence

## Abstract

Amid the global nursing shortage, artificial intelligence (AI) offers a solution to align patient needs with nursing expertise, ensuring equitable workload distribution. Highlighting the transformative potential of artificial intelligence in healthcare, this editorial underscores its revolutionary impact on nursing staffing. By leveraging AI, we can enhance patient outcomes and operational efficiency and reduce staff burnout. However, challenges like data security and job stability arise. It is pivotal to emphasize its optimal integration, engaging nurses in decision-making, rigorous training, and prioritizing data security. This holistic approach ensures that contemporary healthcare practices benefit from AI's capabilities while upholding core values.

## Editorial

Amid a pronounced global nursing shortage [1], the intricacies of nurse staffing and patient assignments have intensified, magnifying care quality and workload balancing challenges. To ensure patient safety and resource efficiency, innovation is paramount. Leading this transformative charge is the integration of artificial intelligence (AI) and machine learning (ML), offering a holistic approach that harmoniously aligns patient needs, nursing expertise, and equitable workload distribution. This editorial discusses the transformative influence of AI on the future of nursing staffing, highlighting its potential to enhance patient assignment processes.

The AI-driven staffing model presents a myriad of opportunities [2]. Its capabilities include delivering data-driven and factual insights, providing real-time and dynamic staffing optimization, and managing intricate factors such as patient conditions and nurse specializations. This results in a balanced workload, reduced staff turnover, diminished burnout, enhanced patient outcomes, and operational efficiency. Yet, the rise of AI can reshape traditional nursing roles, emphasizing the importance of transparency [3]. As AI systems become more integrated into patient care, there's a shift from manual and routine tasks to more complex and analytical responsibilities for nurses. This transition underscores the vital importance of transparency in AI applications. It's essential for healthcare professionals, patients, and stakeholders to understand how AI tools make decisions, especially when these decisions can directly affect patient outcomes. Furthermore, ensuring objective AI outputs and fostering open discussions with nursing staff about AI's role is crucial for trust-building.

Nevertheless, the AI transition is not devoid of challenges. While it can streamline processes and reduce workloads, concerns around job stability, autonomy, and data security can surface. The increased connectivity and reliance on digital tools can threaten patient data privacy [4,5]. Therefore, proactive measures can be taken to mitigate these risks, such as implementing robust cybersecurity protocols, regularly updating software to patch vulnerabilities, and educating nurses about potential threats are essential steps in safeguarding patient information. 

Several steps are imperative to realize the potential of AI-driven staffing while navigating these challenges. These encompass change management, rigorous training, consistent communication, and robust ethical guidelines. By harnessing the power of AI, healthcare institutions can achieve a dual goal: matching nurses with patients to optimize care quality and ensuring the judicious utilization of the valuable nursing workforce.

However, this transformative journey requires a balanced approach. Combining the capabilities of AI with human expertise is paramount to ensure the safety and well-being of both patients and nurses. With robust encryption measures and stringent access controls, AI can be positioned as a transformative tool, reshaping staffing practices and upholding the core values of healthcare organizations.

A holistic strategy is pivotal. Engaging nurses in decision-making, extensive training, and viewing AI as a supportive, collaborative tool are both critical to a smooth transition. Above all, a steadfast commitment to ethics, continuous oversight, and ensuring data security form the cornerstone of successfully integrating AI into nursing staffing.
